# Energy-aware bio-inspired spiking reinforcement learning system architecture for real-time autonomous edge applications

**DOI:** 10.3389/fnins.2024.1431222

**Published:** 2024-09-23

**Authors:** Joshua Ifeanyi Okonkwo, Mohamed S. Abdelfattah, Peyman Mirtaheri, Ali Muhtaroglu

**Affiliations:** ^1^Biomedical Engineering MS Program, Oslo Metropolitan University, Oslo, Norway; ^2^Department of Electrical and Computer Engineering, Cornell University, New York, NY, United States; ^3^Department of Machines, Electronics and Chemistry, Oslo Metropolitan University, Oslo, Norway; ^4^Advanced Health Intelligence and Brain-Inspired Technologies (ADEPT) Research Group, Oslo Metropolitan University, Oslo, Norway

**Keywords:** reinforcement learning, system architecture, spiking neural network, neuromorphic hardware, low-cost, low-energy, context-dependent task, autonomous

## Abstract

Mobile, low-cost, and energy-aware operation of Artificial Intelligence (AI) computations in smart circuits and autonomous robots will play an important role in the next industrial leap in intelligent automation and assistive devices. Neuromorphic hardware with spiking neural network (SNN) architecture utilizes insights from biological phenomena to offer encouraging solutions. Previous studies have proposed reinforcement learning (RL) models for SNN responses in the rat hippocampus to an environment where rewards depend on the context. The scale of these models matches the scope and capacity of small embedded systems in the framework of Internet-of-Bodies (IoB), autonomous sensor nodes, and other edge applications. Addressing energy-efficient artificial learning problems in such systems enables smart micro-systems with edge intelligence. A novel bio-inspired RL system architecture is presented in this work, leading to significant energy consumption benefits without foregoing real-time autonomous processing and accuracy requirements of the context-dependent task. The hardware architecture successfully models features analogous to synaptic tagging, changes in the exploration schemes, synapse saturation, and spatially localized task-based activation observed in the brain. The design has been synthesized, simulated, and tested on Intel MAX10 Field-Programmable Gate Array (FPGA). The problem-based bio-inspired approach to SNN edge architectural design results in 25X reduction in average power compared to the state-of-the-art for a test with real-time context learning and 30 trials. Furthermore, 940x lower energy consumption is achieved due to improvement in the execution time.

## 1 Introduction

Device markets associated with the Internet of Things (IoT) continue to grow relentlessly, fueled by new segments such as the Internet of Bodies (IoB) comprising wearable and implantable intelligent sensor systems. The widespread computation paradigm, which consists of a small cloud-connected embedded system at the edge, is rapidly transitioning to a new model. The edge sensor nodes will have artificial intelligence (AI) for quick and autonomous decision-making with high security (Brainchip and GSA, 2023). However, this is not a simple feat due to the lack of energy-efficient approaches to small-scale recognition and classification problems. The computational effectiveness of the human brain is well-recognized, particularly due to the combined data storage and processing in the same element (Mechonic and Kenyon, 2022). A typical human brain has 10^11^ neurons and 10^15^ synaptical connections, which consume 20 W of power. Considering a digital simulation of a similar artificial neural network consumes 7.9 MW, it is clear novel approaches are needed (Wong et al., 2012). Parallel and event-driven architectures (Dan and Poo, 2004), exemplified by Spiking Neural Networks (SNNs), operate based on Spike-Timing-Dependent Plasticity (STDP). This mechanism facilitates the dynamic adjustment of synaptic connection strengths in response to temporal patterns between presynaptic and postsynaptic spikes.

SNN-based architectures provide a compelling framework as energy-efficient, biologically plausible alternatives to traditional von Neumann machines. Spiking neurons can be modeled with varying complexity (Izhikevich, 2004). Most implementations avoid the full Hodgkin-Huxley model (Hodgkin and Huxley, 1952) that requires solving a minimum of four differential equations with tens of parameters using floating point arithmetic. For example, the Leaky integrate-and-fire (LIF) model (Gerstner and Kistler, 2012) provides a good compromise between accuracy and complexity.

An artificial learning agent in a reinforcement learning (RL) system discovers which actions yield the most reward through trials (Sutton and Barto, 2018). Sequential decision processes in RL are promising for implementing intelligence in autonomous robots. Hence, associated learning algorithms have been under scrutiny. Neuromorphic many core processors, such as Loihi (Davies et al., 2018), have enormous benefits compared to general-purpose processors in addressing machine learning applications at high energy efficiency. Loihi is a neuromorphic processor with an energy-delay product that outperforms conventional CPUs by over three orders of magnitude. It features 16 MB of synaptic memory, enabling over 2.1 million synaptic variables per mm^2^—more than triple the density of its predecessor, TrueNorth. While Loihi's neuron density is slightly lower than TrueNorth's, this trade-off allows for a significantly expanded feature set, including support for various sparse matrix compression models, flexible network connectivity, and variable weight precision. These enhancements ease programming constraints and improve the processor's versatility. Neuromorphic processors are sufficiently flexible and programmable to implement versions of supervised, unsupervised, or reinforcement learning schemes on up to one million neurons per chip. However, such solutions have high costs, complexity, and power dissipation that do not often fit the application-specific optimization requirements of the small edge sensors. Work presented by Donati et al. (2019) utilizes a custom Dynamic Neuromorphic Asynchronous Processor (DYNAP) for executing SNN composed of 192 neurons. The spiking learning method achieves 74% accuracy with SNN power consumption of only 0.05 mW. The component does not have an online learning feature, but provides a promising initial step for application specific real-time low-energy operation.

Field-Programmable Gate Arrays (FPGAs) have been the platform of choice recently to investigate application-specific optimizations in digital SNN architectures. Among these, multiplier-less approaches have received much focus in achieving higher speed with lower cost and power dissipation (Soleimani et al., 2012; Farsa et al., 2019; Asgari et al., 2020). In both (Farsa et al., 2019; Asgari et al., 2020), 32-bit fixed-point numbers represent digitized neuron voltage potential and synapse weights, and simple shift operations replace complex multiplication functions. FPGA implementation in Asgari et al. (2020) builds on recent animal studies with shorter sequences to learn from single stimulus-response pairs across multiple contexts (Raudies and Hasselmo, 2014). Although the size of the RL network required for the application is modest, a synapse module in this work consumes 172 slices of Look-Up Tables (LUTs) and 38 flip-flops (state elements). A digital architecture is targeted in Yang et al. (2020) using compartmental neuron (CMNs) models to enhance biological realism through a multiplier-less approach for energy efficiency. While the implementation on four Altera Stratix III EP3SL340 FPGsA offers significant advancements in biological realism and computational efficiency, it comes with a relatively high network communication overhead for simple tasks. Yang et al. (2022) introduces a biological-inspired cognitive supercomputing system (BiCoSS) that uses large-scale spiking neural networks (SNNs) to study neural mechanisms. BiCoSS features a digital architecture with over four million neurons implemented on FPGAs. It is reported to outperform other large-scale approaches in real-time computational capability, but is not evaluated for smaller scale energy-aware edge applications. NADOL architecture presented in Yang et al. (2024) incorporates dendritic processing to enhance spike-driven learning. NADOL improves power efficiency and learning speed, but has lower accuracy compared to supervised deep learning networks due to limitations of the two-layer architecture.

Different techniques are demonstrated in our previous work (Rasheed et al., 2024) to reduce logic complexity in implementing STDP for reinforcement learning at the edge. Given a context-dependent task with rewarding and non-rewarding action sequences (Raudies and Hasselmo, 2014), significant RL network energy savings are henceforth achieved compared to the state of the art. However, all spikes generated by the network need to be monitored by an external processor, and replay cycles must be activated at the correct times for learning to occur. Context changes must be manually detected, and frequent relearning (replay) activation is necessary to retain memory. Hence, the complete system based on the existing RL network is incompatible with real-time autonomous operation. A challenging aspect of real-time operation is preserving energy efficiency while simultaneously achieving high learning accuracy. Not an easy feat, this is achievable through bio-inspired enhancements at the system architecture level. These enhancements are outlined below as the original contributions of this work and are further detailed in the following sections.

i) *Replay with synapse locking*: Recent animal studies indicate synapses can saturate after Long Term Potentiation (LTP) and Long Term Depreciation (LTD) activities (Nguyen-Vu et al., 2017). This results in temporary inhibition of further LTP and LTD activities on the same synapses, which can also be considered a desirable property for energy-efficient RL architectures. As long as the context does not change, synapses with accumulated “large” weights through LTP or “small” weights through LTD can exploit their recent learning instead of modifying their weights further during the next exploration cycles. The Replay Sequencer proposed in this work contains a synapse locking mechanism that will disable the synapses with extremely elevated or diminished weights through recent LTP/LTD operations from changing until the detection of the next context change.
ii) *Senso-motoric event detection and scratchpad*: Our previous implementation (Rasheed et al., 2024) includes a scratchpad to add a temporal hysteresis to hippocampus neurons to prevent frequent LTP/LTD activities on the same mid-layer neuron, which consequently optimizes the overall RL energy dissipation. Further enhancements are proposed in this work to save energy associated with LTP/LTD events, inspired by a relationship discovered two and a half decades ago between synaptic tagging and long-term potentiation (Frey and Morris, 1997). A Senso-Motoric Event Detection Unit and a new Scratchpad Unit are integrated to identify spike sequences that represent a new context or new Senso-motoric aspects of an existing context. Temporal hysteresis is then applied to prevent modification of synapse connections unrelated to such significant events. This ensures that recurring senso-motoric observations during exploration do not create new energy-consuming replay cycles.
iii) *Learning evaluation and exploration reseeding*: Recent findings not only stress the significance of insula and ventromedial prefrontal cortex (vmPFC) in balancing exploration and exploitation in reinforcement learning (Blanchard and Gershman, 2017), but infer a monitoring function at vmPFC and a response function at dorsomedial prefrontal cortex (dmPFC) for the ongoing action plan to drive new exploration schemes (Domenech et al., 2020). This phenomenon inspires a Learning Evaluation Unit in the presented hardware organization.
iv) *Power management through coarse and fine clock gating*: Patterns of localized brain activation have long been observed during cognitive tasks (Posner and Raichle, 1994). It has also been claimed that spatially localized brain activation can be regulated voluntarily through learning (deCharms et al., 2004). Both coarse and fine clock gating features integrated into the power management architecture in this work serve the same purpose as containing the power dissipation to the portions of the hardware that need to be essentially active. Clock gating is a well-known power reduction technique in digital electronics. Nevertheless, the particular application of the scheme in this work properly complements the rest of the presented bio-inspired approaches to reduce energy consumption further.

The rest of the paper is organized as follows: Section 2 presents an energy-aware implementation of the bio-inspired real-time system architecture for the context-dependent RL task with the features described above in (i–iv). Section 3 presents verification and simulation results to quantify the benefits of the new architecture. Finally, a discussion of the results is provided and significant conclusions are summarized.

## 2 Materials and methods

### 2.1 Existing framework

**Figure 1:**
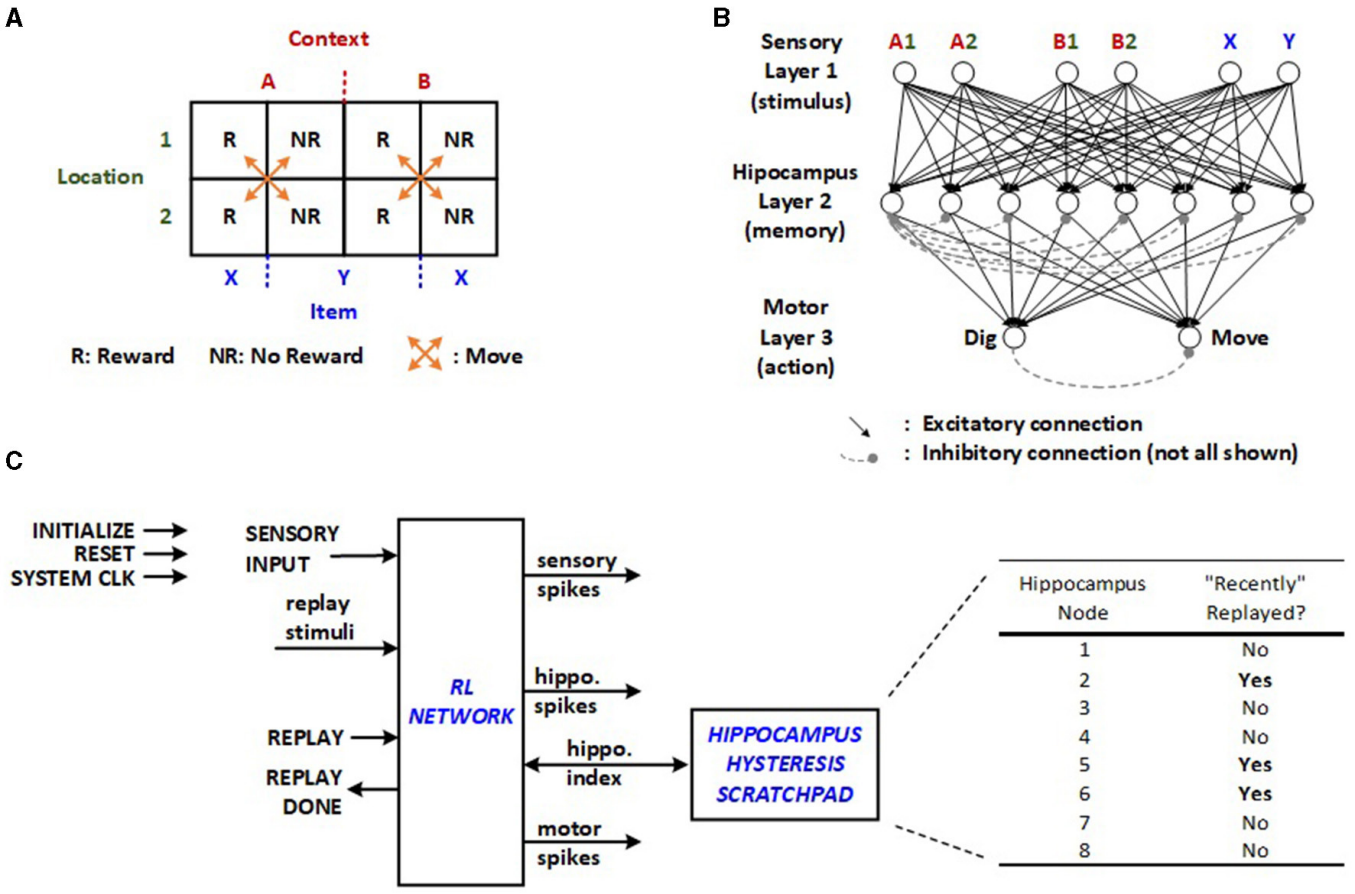
**(A)** Context-dependent task, **(B)** SNN for the given RL task (Raudies and Hasselmo, 2014), and **(C)** original RL network implemented using SystemVerilog on FPGA with off-line learning control and hippocampus hysteresis scratchpad (Rasheed et al., 2024).

A relatively straightforward RL model for a context-dependent task is chosen for the edge application based on Raudies and Hasselmo (2014). This task involves a total of four physical locations, with two in context A (A1, A2) and two in context B (B1, B2), as illustrated in Figure 1A. Each location contains either item X or Y, resulting in eight possible triplets (e.g., A1X, A2X, A1Y, etc.). These triplets correspond to the activation of different pairs of sensory inputs in the SNN, as displayed in Figure 1B. Upon selecting between the two contexts and a location within the chosen context, the mouse receives a reward for exactly half of these triplets. When activations from one pair of sensory neurons move through the second layer to the third, the motor neurons generate a “dig” or “move” action. The inhibitory interconnections among neurons in the hippocampus and motor layer, depicted with dashed lines in Figure 1B, facilitates the winner-take-all (WTA) network as described in Asgari et al. (2020). To achieve this, inhibitory non-plastic synapses with substantial negative weights are incorporated into the network. In the proposed structure, each inhibitory connection is realized through a cost-effective combinational dendrite link with a fixed negative value, activated by the first neuron to fire in a given layer. It is anticipated that multiple neurons in a layer may fire simultaneously as the supported number resolution is decreased for optimizing energy. Therefore, the proposed scheme implements prioritization, which is configurable via multiplexed inhibition wires as part of the network's random initialization. Incorporating random WTA priority decreases the latency of the exploratory RL trials.

The machine learning algorithm involves repeated cycles of exploration and replay modes orchestrated by an external controller or software in the original approach. During the first phase, random triplets are produced to simulate exploratory trials by feeding sensory input in Figure 1C. The prosecution can involve multiple “move” motor actions before culminating in a “dig”, transitioning the machine to the replay mode or the second phase. Based on whether the “dig” yields a reward, the network undergoes either potentiation or depression of the synapses that contribute to the final “move” and “dig”. This necessitates the external controller to monitor all related neuronal activity.

The random initialization of neuron potentials and synapse weights may lead to numerous “move” iterations in the exploration phase before any replay can occur for learning, inevitably lengthening the learning latency. Furthermore, the same synapses may undergo both potentiation and depression operations across consecutive replays. Although this is expected to correct its course over time, it wastes time and energy. The previously proposed implementation with off-line learning integrates a hippocampus hysteresis scratchpad, as illustrated in Figure 1C, which monitors whether the synapses from the last sequence have been recently altered. If they have, the replay cycles are bypassed. Once the scratchpad indicates all hippocampus nodes have been recently replayed, the same synapses can be adjusted again, allowing plasticity to take effect after fully utilizing the existing hippocampus neurons.

### 2.2 Senso-motoric event detection and scratchpad

**Figure 2:**
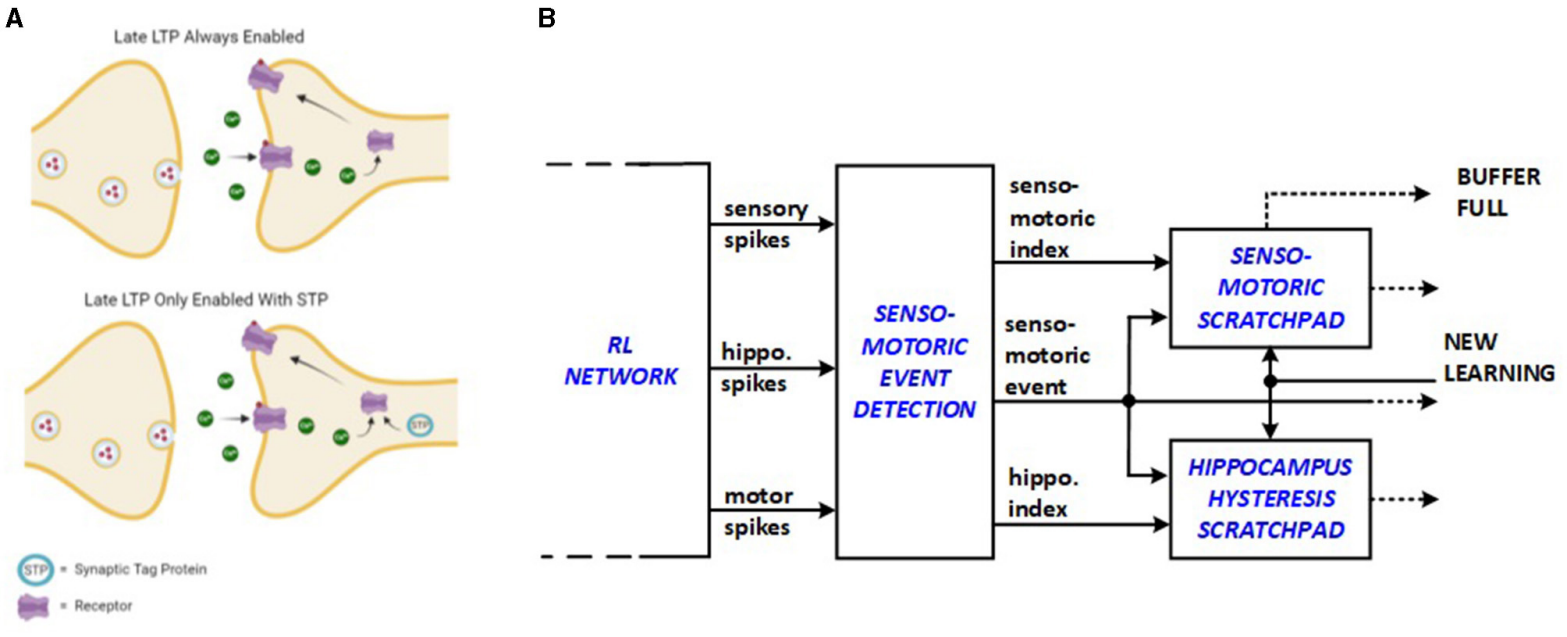
Bio-inspiration from **(A)** synaptic tagging for new hardware units: **(B)** senso-motoric event detection and scratchpad.

It is proposed by Frey and Morris (1997) that long-term potentiation (LTP), which is primarily responsible for memory in the mammalian brain, initiates the creation of short-lasting “synaptic tag” at the potentiated synapse to delay the next LTP event as shown in Figure 2A. This behavior is expected to save energy because the repetitive stimulation of the same synapses for new learning processes is avoided. Senso-motoric activity of the network is monitored in this work such that input triplet-output dig/move groups are suppressed from relaunching new energy-consuming replay sequences through the added hardware units. Bio-inspired senso-motoric event detection and scratchpad features are depicted in Figure 2B as an enhancement to the previous hippocampus hysteresis scratchpad. The senso-motoric hardware implementation of the system effectively manages sensory inputs, events, and rewards by tracking various combinations of valid triplets, corresponding actions, and rewards, preventing redundant replay events. The scratchpads help detect context changes by identifying new reward feedback for different events, ensuring that only relevant data is processed. A synapse saturation signal indicates when a context is fully explored, and if a context change is detected, the scratchpads update with new values.

The senso-motoric implementation saves energy by optimizing how sensory inputs, events, and rewards are processed. The synapse saturation signal indicates when a context has been fully explored, signaling that learning in that context is complete. If a context change is detected, the scratchpads are updated with new values, ensuring the system only focuses on new, relevant information, which further minimizes energy use.

### 2.3 Learning evaluation and exploration reseeding

**Figure 3:**
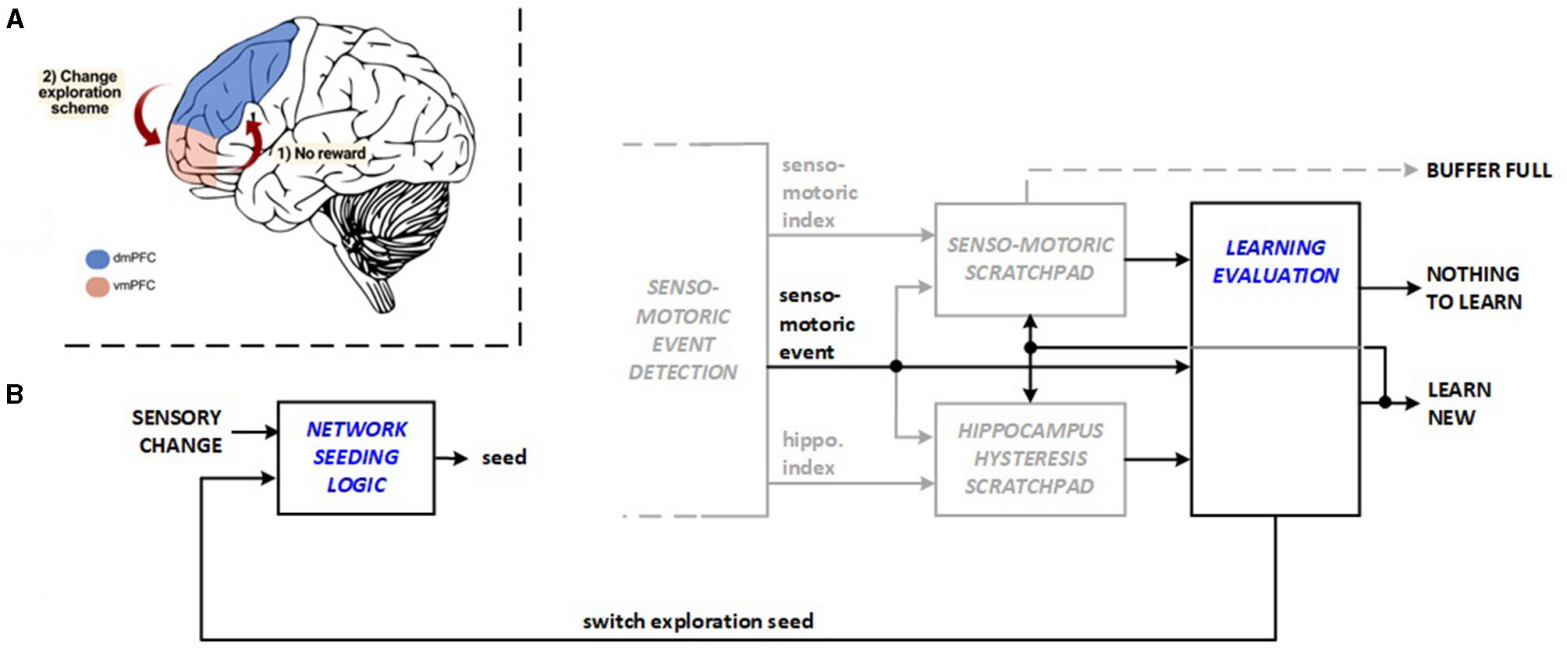
Bio-inspiration from **(A)** dmPFC and vmPFC coordination for changing exploration scheme for new hardware units: **(B)** learning evaluation and network seeding logic.

Exploring new action plans vs. exploiting previous learning requires a delicate balance, because new explorations and new replays for learning consume energy. Recent studies identify collaboration between the insula and ventromedial prefrontal cortex (vmPFC) in balancing exploration and exploitation in reinforcement learning (Blanchard and Gershman, 2017). vmPFC monitors the ongoing plan while dorsomedial prefrontal cortex (dmPFC) exhibits activation for new exploration when the existing plan is not rewarding (Domenech et al., 2020), as illustrated in Figure 3A. The Learning Evaluation unit, designed to achieve a more energy-efficient balance in the machine, serves two purposes: The unit skips learning (replay) activities when exploration does not result in new material to learn. It also triggers exploration reseeding in this scenario and in cases when there has been a long exploration period without any discovery. This operation changes the network stimulus randomization seed and the inhibition priority across the hippocampus layer to reduce the time needed to identify the subsequent meaningful discovery during exploration. Figure 3B depicts bio-inspired learning evaluation and exploration of reseeding features. The transitions between bio-inspire learning evaluation and exploration reseeding are managed by the reinforcement learning (RL) system control module running in the FPGA hardware, which functions as a Moore-type Control Unit. The hardware architecture manages transitions between system states (initialization, rest, explore, and replay) to optimize energy use. It activates learning and replay processes only when needed, avoiding unnecessary activities, which reduces energy consumption.

The energy savings within the system are achieved through efficient management of transitions between different states (system initialization, system rest, explore, and replay) by the reinforcement learning (RL) system control module. The system operates across defined states, ensuring that energy-intensive processes like learning and replay are only activated when necessary, avoiding unnecessary exploration or learning activities that would otherwise consume additional energy.

### 2.4 Replay with synapse locking

**Figure 4:**
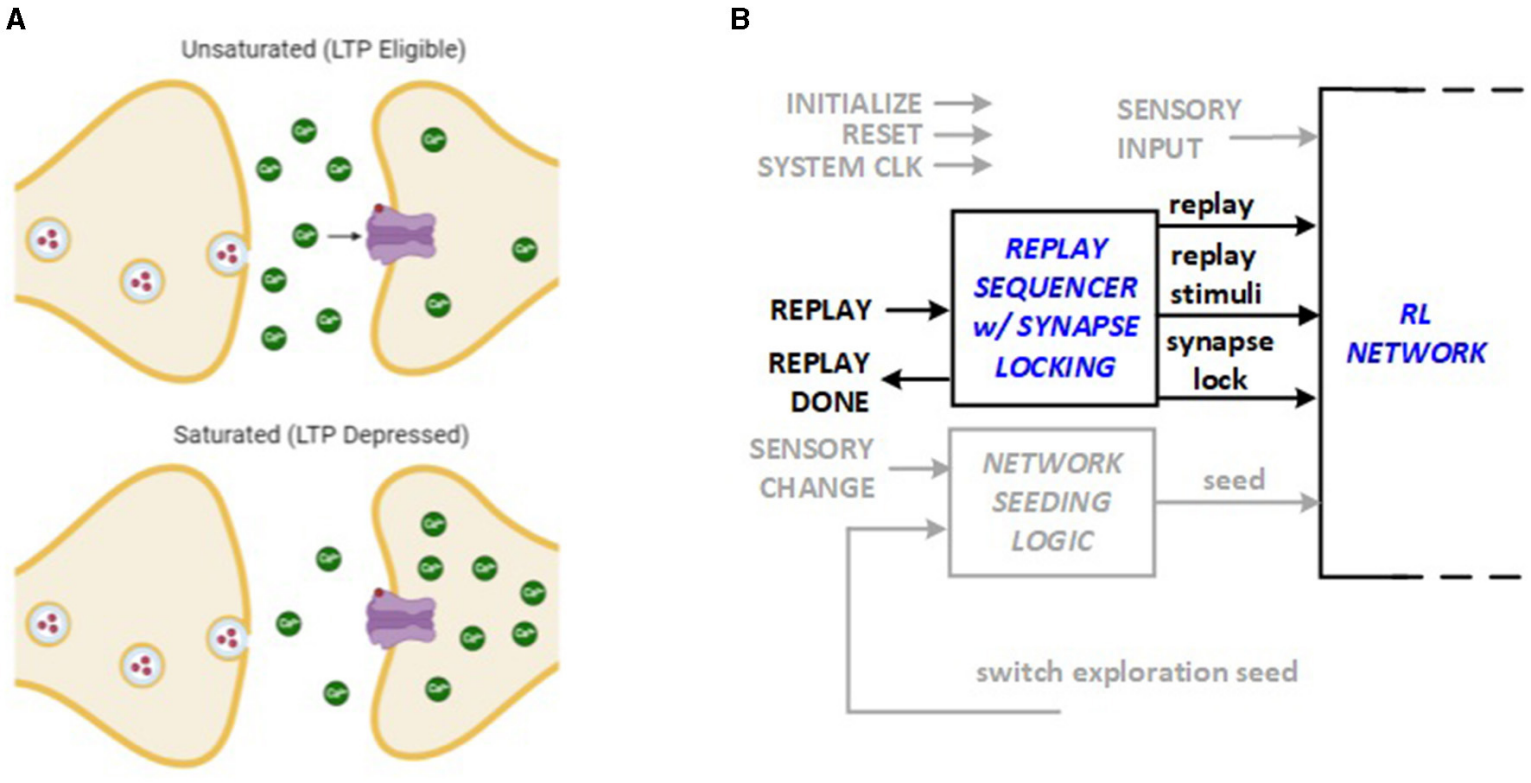
Bio-inspiration from **(A)** synapse saturation for new hardware feature: **(B)** synapse locking as part of Replay Sequencer Unit.

The saturation hypothesis explains how synapse can temporarily inhibit further LTP and LTD activities based on recent history of experience (Nguyen-Vu et al., 2017). The idea, illustrated in Figure 4A, is utilized in this work to lock each synapse weight after the replay sequence that modifies it. The lock stays in effect until a discrepancy is discovered in reward patterns, for example, due to a context change, which causes the synapse to unlock for the subsequent replay. This enhancement to the hardware architecture requires a “lock” bit to be stored in each synapse. The locking and unlocking mechanism is integrated into the Replay Sequencer Unit as shown in Figure 4B. The RL system control module running in the FPGA hardware includes a replay sequencer using a Johnson counter to revisit previous experiences and strengthen learning. It sequences neurons and applies replay signals to support synaptic plasticity (LTP and LTD). The synapse lock function ensures that specific synaptic connections are preserved or modified as needed, and targeted neurons are activated during replay to reinforce learning.

The system conserves energy by using a replay sequencer to revisit only necessary past experiences, reducing computational load. It applies replay signals selectively to essential neurons, ensuring efficient synaptic plasticity without unnecessary adjustments. The synapse lock feature stabilizes key connections, preventing continuous changes, and only activates neurons needed for replay, minimizing energy use.

### 2.5 New energy-efficient RL datapath architecture

**Figure 5:**
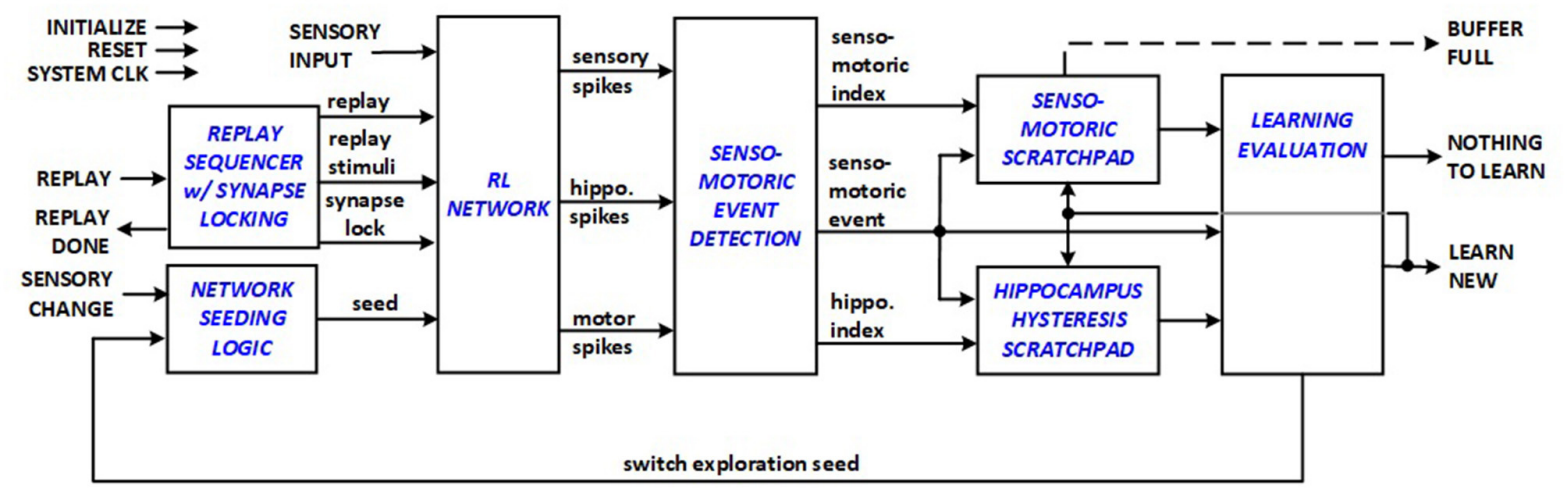
RL system datapath.

The entire datapath architecture for energy-efficient RL systems to support edge applications is depicted in Figure 5, which presents the datapath of the implemented top-level module, and comprises sensory, hippocampus and motor neurons organized in a *RL network* structure designed to associate sensory inputs with motor actions through reinforcement learning. The system is equipped with various input and output signals for operation control and interaction with external system components, including initialization, reset, system clock (CLK), sensory input (sense), and reward signals. In the presented framework, reward corresponds to digging that results in finding the seeked item e.g., cheese for a mouse. The datapath evaluates if a new senso-motoric discovery is made during exploration phase, and signals either *NOTHING TO LEARN* or *LEARN NEW* to indicate to the system control unit if a new replay sequence needs to be started for learning. In turn the control unit asserts *REPLAY* signal to start a new learning session. Network seeding logic allows variations in initialization of synapses and neurons and prioritization order in WTA scheme.

The RL system datapath thus integrates sensory triplet detection, filtering, encoding, and staging logic that allow the RL network to function with autonomy. The RL system control module manages state transitions and control signals essential for the RL system's operation. The RL Network module is tailored for synchronous reinforcement learning, with multiple interconnected neuron layers that process sensory inputs, generate spikes, and produce motor responses. Each neuron node module goes through different states (active, waiting) before qualifying for firing. Each synapse module represents the synaptic connections between neurons in the network, maintaining and modifying synaptic weight as a result of LTP/LTD operations.

Comparing this design and the starting architecture in Figure 1, the hardware complexity is inevitably increased. However, the added complexity is well-justified, because the initial approach does not have fully autonomous learning capabilities in real-time. Off-line learning requires a minimum of support for an additional external processor, which generally increases both cost and average power dissipation. The following section will explore the theoretical and simulation-based estimation of the benefits associated with the features supported by the new architecture.

### 2.6 Power management

#### 2.6.1 Coarse clock gating in control unit

**Figure 6:**
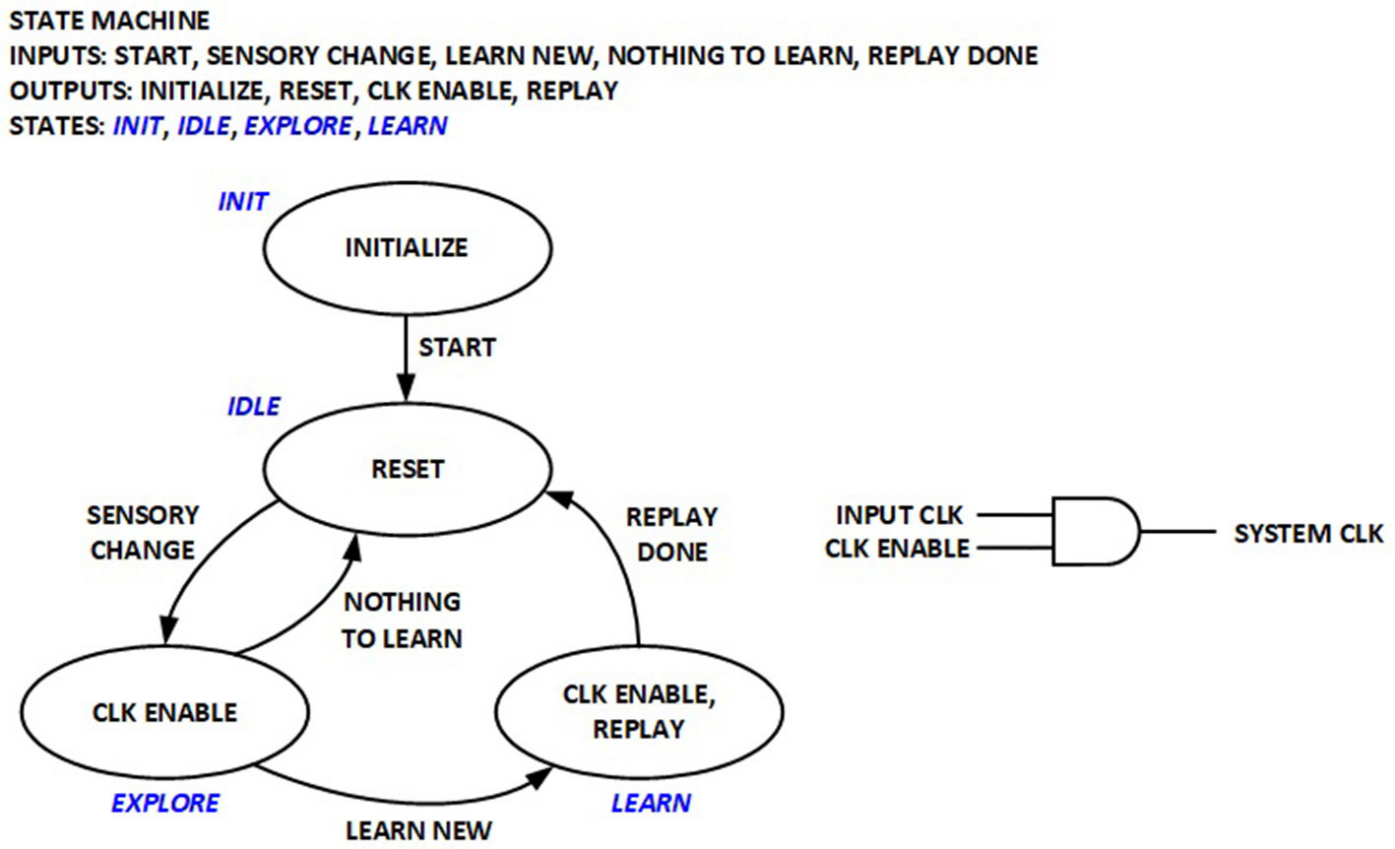
Finite-State Machine (FSM) for real-time RL control and coarse clock gating.

A simple control Finite State Machine (FSM) is implemented to orchestrate various operation phases in the previous implementation for real-time RL, as depicted in Figure 6. The System clock used to run the datapath logic is only enabled during exploration and learning phases, but is otherwise turned off. This implementation significantly reduces average power by eliminating dynamic power consumption in the datapath logic for edge sensors where new sensory inputs may not be available for long periods. The RL processor is thus compatible with the duty cycling power management modes widely utilized in wireless sensor networks.

#### 2.6.2 Fine clock gating in synapse logic

**Figure 7:**
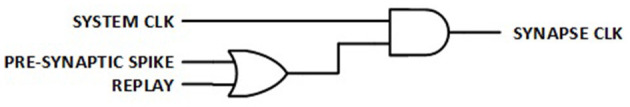
Fine clock gating per synapse.

Synapses are the most common logic elements in RL network. Therefore, turning off the individual clock signal to each synapse when there is no pre-synaptic spike to process and no replay, as shown in Figure 7, results in a significant reduction in dynamic power consumption.

## 3 Results

Full RL system architecture, which consists of the datapath and control units illustrated in Figures 5, 6, respectively, is implemented using SystemVerilog hardware description language and synthesized for Intel MAX10 Dual-Supply FPGA 10M08DAF256C8GES. A simulation testbench is utilized to evaluate the time it takes to explore and discover all possible triplet combinations from the sensor inputs. A replay phase is launched once the exploration results in a rewarding or non-rewarding dig action to train the associated neurons. After all triplet combinations are discovered, 100 trials of random sensory inputs are executed to investigate RL accuracy. After the testbench detects 100 occurrences of motor (dig or move) events, half of the rewarding outcomes are changed in the context, and 100 more random trials are run to verify the system's ability to adapt to changes in context.

### 3.1 Senso-motoric event detection and synapse locking

**Figure 8:**
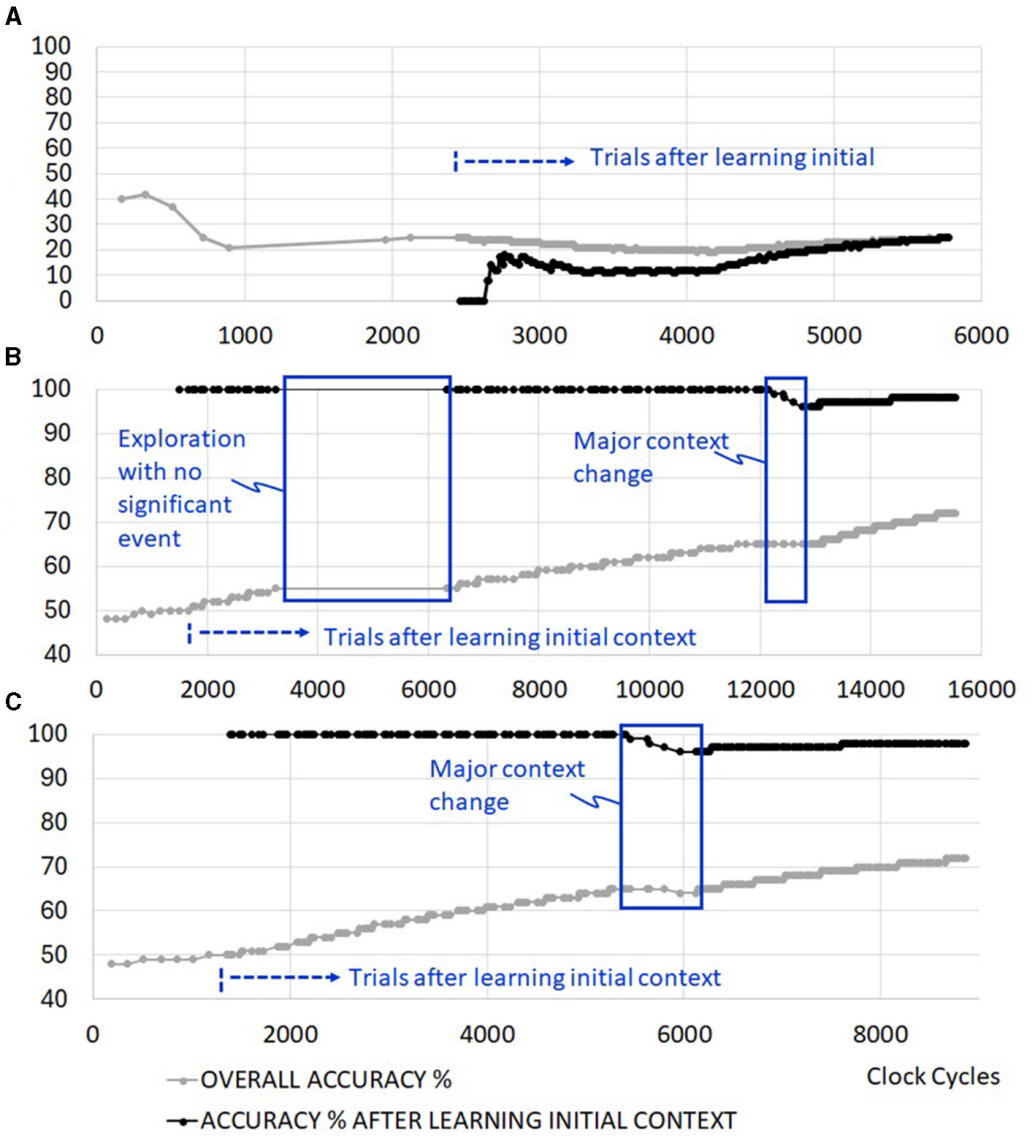
Example simulation results with **(A)** no real-time autonomous learning features (requires a control processor support), **(B)** senso-motoric event detection and synapse locking features *without* exploration reseeding, **(C)** senso-motoric event detection and synapse locking *with* exploration reseeding.

Figure 8A depicts the system with no real-time autonomous bio-inspired learning features. The system is able to discover different sensory scenarios to learn, but is unable to retain the learning without support from an external processor. Figure 8B excludes exploration reseeding and clock gating features. However, senso-motoric event detection and synapse locking are included to retain short and long-term memory of observed rewards. Once the system trains itself for the current context in about 1,350 clock cyles, 100% accuracy is achieved for all triplets afterwards. There are delays in resolving new sensory events during the trial runs. This is marked in Figure 8B as the period of exploration with no significant event. Due to a lack of reseeding in the network, the system cannot generate meaningful sensory and motoric spike sequences during this period. Once the significant context change occurs in cycle 12,250, the system retrains itself to slowly approach 100% accuracy again. Overall accuracy in the figure includes the firing of non-rewarding motor actions during the learning phase. When the simulation cycles are extended, initial errors become negligible, and this curve approaches 100% as well.

### 3.2 Addition of exploration reseeding

Exploration reseeding allows faster resolution of different sensory inputs in the network, as shown in Figure 8C. There are 100 trials before and 100 trials after context change. The operations, which last up to 15,500 cycles (Figure 8B) when learning evaluation with exploration reseeding is not available, complete by clock cycle 8,870. As will be further discussed later, this results in significant energy reduction in the operation of the edge RL system.

### 3.3 Power management through clock gating

**Figure 9:**
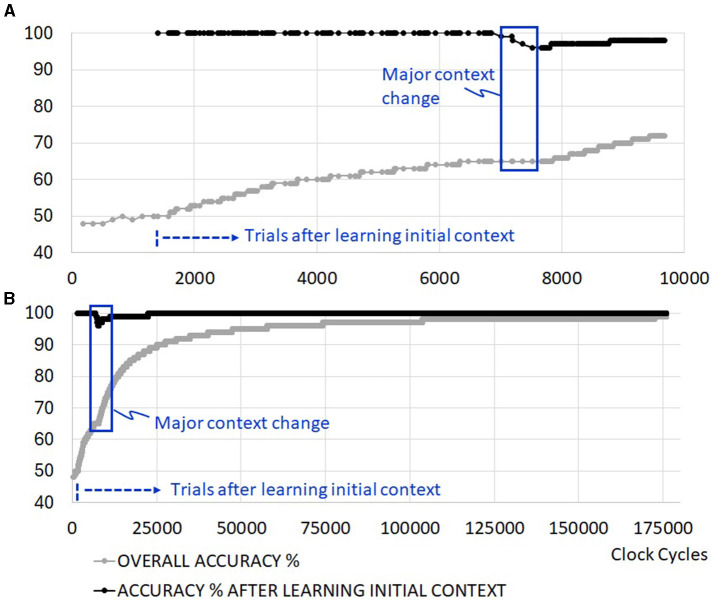
Example simulation with added clock gating. **(A)** First ~10,000 cycles, including one major context change, **(B)** convergence to 100% in the absence of further context changes.

The learning time of the initial context does not extend with coarse clock gating, but the total execution time of running the trials is extended as depicted in Figure 9A. This is because the evaluation of new sensory inputs in the RL network is disrupted with clocks turning on and off as the machine switches between states. One would need to investigate the average power reduction benefit of the coarse clock gating against the extended execution time in scenarios with more realistic idle time to determine the impact on energy consumption from this feature fully. Fine clock gating at the level of synapses does not add any penalties to the execution time. Figure 9B illustrates an example of an extended set of trials where the overall accuracy approaches 100% as learning (training and retraining) periods become negligible.

### 3.4 Quantification of feature benefits

MAX10 Dual-Supply FPGA supports a core supply voltage of 1.2 V for lower power consumption. Different design versions comfortably run at 62.5 MHz clock frequency at this supply voltage. Sleep modes available on this FPGA have not been turned on, but can be utilized in energy-aware applications where the RL network stays idle for extended periods. Therefore, the comparisons in Table 1 only concern active modes of operation.

**Table 1:** Simulated feature analysis of cost, execution time, average power, and energy dissipation.

**Design metric**	**Baseline^*a*^ no autonomy**	**SED & SL^*b*^**	**SED & SL + ER^*c*^**	**AF & CCG^*d*^**	**AF & CFCG^*e*^**
Cost (No. of LEs /registers)	2,706/630	4,574/948	4,587/948	4,564/948	4,731/1050
Accuracy (%) after	12	100	100	100	100
init. context learning					
Execution time (μs)	39.2	21.6	21.8	21.8	21.8
init. context learning					
Execution time (μs)	66.2	192.5	84.6	108.7	108.7
with 100 trials					
Power cons. (*mW*)	21.59	44.9	43.81	41.73	30.04
Energy cons. (*nJ*)	846	968	954	909	655
init. context learning					
Energy Cons. (*nJ*)	1,430	8,644	3,706	4,536	3,265
with 100 trials					

^*a*^Logic-optimized (Rasheed et al., 2024) with no real-time learning features.

^*b*^Senso-motoric event detection and synapse locking.

^*c*^Sensomotoric event detection and synapse locking + exploration reseeding.

^*d*^All previous features + coarse clock gating.

^*e*^All previous features + coarse and fine clock gating.

The cost of implementation grows with added features, as expected. Real-time self-driven implementation of a 26-node RL network roughly doubles compared to the simple (baseline) RL network with no online autonomous learning capacity (first column in Table 1). Energy reduction features add to implementation cost by about 5%. Clock cycle count during the learning of initial context reduces significantly with added bio-inspired features, as observed in the third row of the table. However, resolving of sensory inputs for determining action takes much less time when there is support for exploration reseeding (fourth row of the table). The exploration reseeding feature reduces average power consumption due to reduced switching activity. However, the actual impact on power dissipation comes with the fine-grain clock gating feature represented by the last column of Table 1. The coarse clock gating feature does not reduce energy consumption due to increased execution time while running trials, although energy consumption is reduced during learning activity. As the idle time between consecutive sensory inputs increases, coarse clock gating may start showing further advantage. Energy consumption of the baseline during the 100 trials should be ignored due to extreme inaccuracy in resolving the sensory inputs in the absence of an external processor support. Implementing fine clock gating improves energy consumption by 33%.

## 4 Discussion

When the learning is finished, (locked) synapses disable local clock switching. Therefore, the fine clock gating feature contributes to energy efficiency during the trial runs. However, as noted in Table 1, other features also contribute to improvement of different design “goodness” measures such as context learning execution time (event detection and synapse locking), execution time after learning during trial runs (exploration reseeding), and power dissipation during context learning (coarse clock gating).

The optimized real-time and stand-alone RL system implementation is summarized in Table 2, in comparison to state of the art, to provide insights into the benefits of the features presented in this work. The proposed system architecture enables fully autonomous reinforcement learning to detect and learn context changes. This is functionally equivalent to the work by Asgari et al. (2020), whereas our previous work (Rasheed et al., 2024) requires an external processor to control the RL network. Special energy-saving synapse features such as locking/unlocking and fine clock gating significantly increase the synapse cost regarding flip-flop and LUT count compared to Rasheed et al. (2024). However, the power-managed synapse cost is still lower than that reported by Asgari et al. (2020). In this work, the cost of supporting an energy-optimized, fully stand-alone real-time RL system is roughly doubled when compared to the simple RL network driven by an external processor (Rasheed et al., 2024). Added energy-saving features degrade the speedpath in this work, which explains the lower clock frequency. However, significantly lower average power dissipation combined with reduced clock cycle count results in significant energy saving that is 940x lower than the one reported by the more complex system in Asgari et al. (2020), and is less than half of our previous non-autonomous implementation (Rasheed et al., 2024).

**Table 2:** Comparison across recent synaptic strength based STDP learning rule implementation for 16-node reinforcement learning system.

	**Asgari et al. (2020)**	**Rasheed et al. (2024)**	**This work**
Autonomy	RT + SA^α^	External control	RT + SA^α^
FPGA used in implementation	Kinetix7	Cyclone IV GX	MAX10 DS^β^
Process technology (*nm*)	28	60	55
Core supply voltage (*V*)	1	1.2	1.2
Synapse cost (*F/L^δ^*)	38/172	6/24	12/57
RL system cost (*F/L^δ^*)	8,096/19,059	538/2,648	1,050/4,731
Synapse clock, *f*_*max*_ (*MHz*)	151	355	126
RL system clock, *f*_*max*_ (*MHz*)	148.4	71	62.5
Cycle count^γ^, *CC*	30,000	2,000	1,887
Average power consumption^γ^ (*W*)	1.81	0.198	0.078
Energy consumption^γ^ (*μJ*)	2,257	6	2.4

^α^RT, real time; SA, stand-alone; ^β^DS, dual supply; ^δ^*F* =# slice flipflops; *L*:# slice LUTs.

^γ^Task includes initial context learning + 30 trials.

## 5 Conclusions

Next edge sensor systems will have simple recognition intelligence integrated to their operations. Although neuromorphic hardware designs can step up to the task, many general purpose neuromorphic processors do not meet the energy budgets of wireless sensors. This research demonstrates that there are many opportunities for inspiration from neurosciences that can help optimize hardware architectures to achieve far better energy efficiency than the state of the art. We particularly focus on four biological features, namely synapse locking, synaptic tagging, change of exploration schemes and localized activation with energy conservation perspective. Integration of these ideas directly to an existing RL network implementation on an FPGA provides an order of magnitude average power savings and close to three orders of magnitude energy savings in performing the task. The presented architecture can potentially be scaled to a custom VLSI design to fit into the microWatt sensor system. Most importantly, the research results reveal brain-inspired computation ideas should be revisited with a new energy-awareness perspective to address the particular needs of real-time edge AI in wireless sensor nodes. The future work will include more complex learning benchmarks for pattern recognition applications.

## Data Availability

The raw data supporting the conclusions of this article will be made available by the authors, without undue reservation.
